# Effect of *Clostridium perfringens* *β*-Toxin on Platelets

**DOI:** 10.3390/toxins9100336

**Published:** 2017-10-24

**Authors:** Anne Thiel, Helga Mogel, Julia Bruggisser, Arnaud Baumann, Marianne Wyder, Michael H. Stoffel, Artur Summerfield, Horst Posthaus

**Affiliations:** 1 Department of Infectious Diseases and Pathobiology, Institute of Animal Pathology, Vetsuisse Faculty, University of Bern, 3012 Bern, Switzerland; anne.thiel@vetsuisse.unibe.ch (A.T.); julia.bruggisser@vetsuisse.unibe.ch (J.B.); marianne.wyder@vetsuisse.unibe.ch (M.W.); 2 Institute of Virology and Immunology, 3012 Bern, Switzerland; arnaud.baumann2@gmail.com (A.B.); artur.summerfield@ivi.admin.ch (A.S.); 3 Department of Infectious Diseases and Pathobiology, Vetsuisse Faculty, University of Bern, 3012 Bern, Switzerland; 4 Division of Veterinary Anatomy, Vetsuisse Faculty, University of Bern, 3012 Bern; Switzerland; helga.mogel@vetsuisse.unibe.ch (H.M.); michael.stoffel@vetsuisse.unibe.ch (M.H.S.)

**Keywords:** *Clostridium perfringens* type C, beta-toxin, platelets

## Abstract

*Clostridium perfringens* *β*-toxin (CPB) is the major virulence factor of *C.* *perfringens* type C causing a hemorrhagic enteritis in animals and humans. In experimentally infected pigs, endothelial binding of CPB was shown to be associated with early vascular lesions and hemorrhage but without obvious thrombosis of affected vessels, suggesting altered hemostasis in the early phase of the disease. The objective of the present study was to investigate the effect of CPB on platelets, with respect to primary hemostasis. Our results demonstrate that CPB binds to porcine and human platelets and forms oligomers resulting in a time- and dose-dependent cell death. Platelets showed rapid ultrastructural changes, significantly decreased aggregation and could no longer be activated by thrombin. This indicates that CPB affects the physiological function of platelets and counteracts primary hemostasis. Our results add platelets to the list of target cells of CPB and extend the current hypothesis of its role in the pathogenesis of *C. perfringens* type C enteritis.

## 1. Introduction

*Clostridium perfringens β*-toxin (CPB) is a soluble 35 kD protein and belongs to the haemolysin family of *β*-pore forming toxins (*β*-PFTs) [1,2]. It is the major virulence factor of *C. perfringens* type C [3], which causes an acute necro-hemorrhagic enteritis in animals and humans. CPB forms oligomeric pores in different neoplastic human immune cell lines [4,5,6] and endothelial cells [7,8]. CPB is highly toxic to porcine and human endothelial cells [9,10] and induces a disruption of the cytoskeleton, the retraction of the cell borders, and necrotic cell death [8]. As opposed to endothelial cells, porcine small intestinal epithelial cells are unaffected by CPB, even at high concentrations [11]. Physiologically, endothelial injury initiates primary hemostasis by exposing platelets to subendothelial matrix, including von Willebrand factor (vWF) and collagen, which leads to binding and activation of platelets. Activated platelets undergo rapid morphological changes and release pro-thrombotic and pro-inflammatory mediators from their granules, which promotes the recruitment of further platelets, adhesion and primary hemostasis [12,13,14]. In end-stage lesions of spontaneously diseased pigs, CPB can consistently be demonstrated on endothelial cells of thrombosed intestinal mucosal vessels [15]. Interestingly, in experimentally induced lesions in piglets, Schumacher et al. [16] observed endothelial binding of CPB, which was associated with early vascular lesions such as edema and extravasation of erythrocytes but without obvious thrombosis of affected vessels. Thus, despite early endothelial targeting by CPB, thrombus formation seems to be a late event in the disease and the progressive hemorrhage might be favored by simultaneous anti-thrombotic effects. We hypothesized that besides endothelial cells, CPB also targets platelets and that a direct toxic effect on these blood components could contribute to the progressive tissue hemorrhage observed in *C. perfringens* type C enteritis. The objective of this study, therefore, was to investigate the effect of CPB on porcine and human platelets.

## 2. Results

### 2.1. Cytotoxicity of rCPB and Morphological Effects of rCPB on Platelets

**Figure 1:**
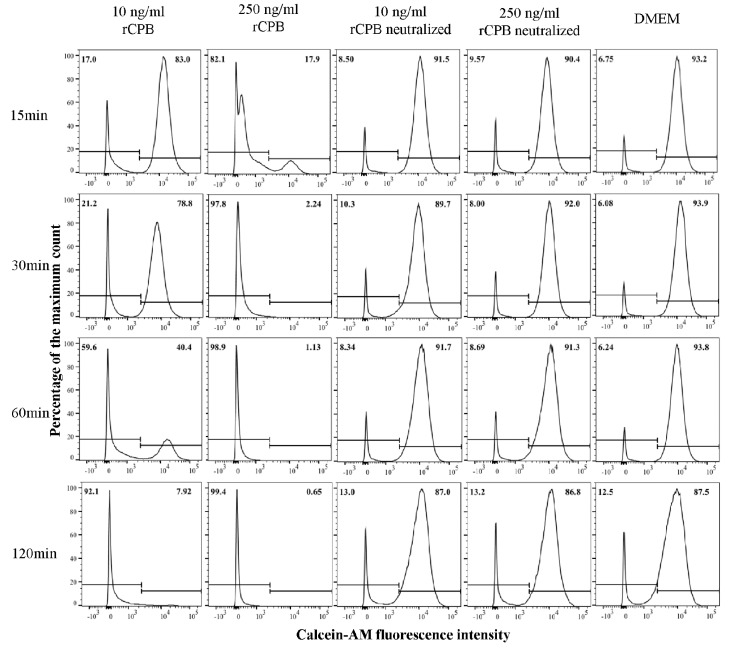
Detection of recombinant *Clostridium perfringens β*-toxin (rCPB)-induced rapid cell death in porcine platelets by flow cytometry. Platelets (33.3 × 10^6^/mL) were incubated with rCPB (10 and 250 ng/mL) or neutralized rCPB for indicated time periods. Viability staining was performed using Calcein-AM (1 μM). Platelets in medium without toxin served as negative control. The histograms show the Calcein-AM fluorescence intensity of platelets, in which the Calcein-AM positive fraction represents viable platelets. Percentages of platelets are indicated in the top of each panel.

**Figure 2:**
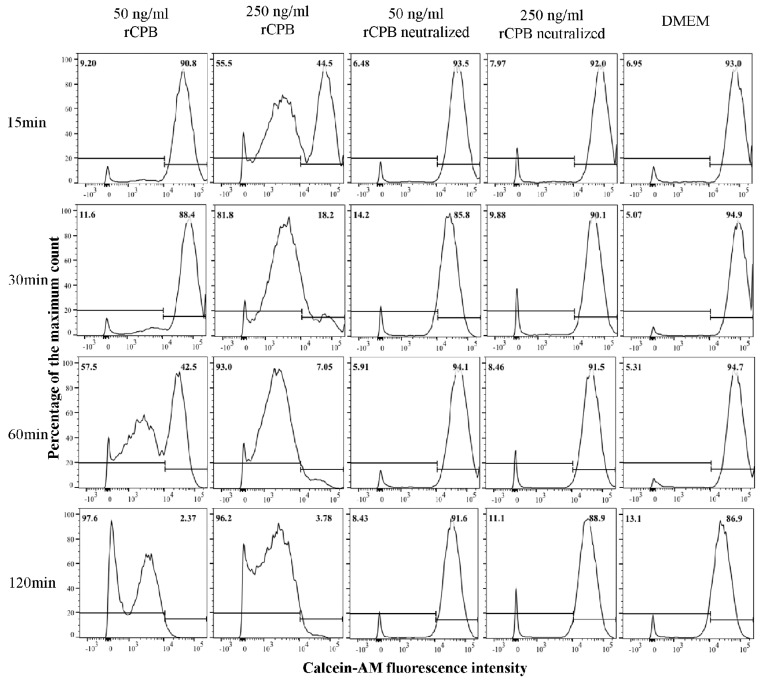
Detection of rCPB-induced cell death in human platelets by flow cytometry. Platelets (33.3 × 10^6^/mL) were incubated with rCBP (50 and 250 ng/mL) or neutralized rCPB for indicated time periods. The viability staining was performed using Calcein-AM (1 μM). Platelets in medium only without toxin served as negative control. Percentages of positively and negatively labeled platelets are indicated in the top of each panel.

Incubation of porcine and human platelets with recombinant CPB (rCPB) resulted in a time and dose-dependent reduction of platelet viability (Figure 1 and Figure 2). Platelets showed a rapid loss of Calcein-AM fluorescence when incubated with 250 ng/mL rCPB. A marked effect was already detected at 15 min of incubation and the majority of platelets had lost Calcein-AM fluorescence after 30 min of toxin incubation. Cytotoxic effects in porcine and human platelets were detectable at very low toxin concentrations of 10 ng/mL (porcine, Figure 1) or 50 ng/mL (human, Figure 2) rCPB. Control incubations with neutralized rCPB or DMEM (Dulbecco's Modified Eagle's Medium) did not affect the viability of platelets.

**Figure 3:**
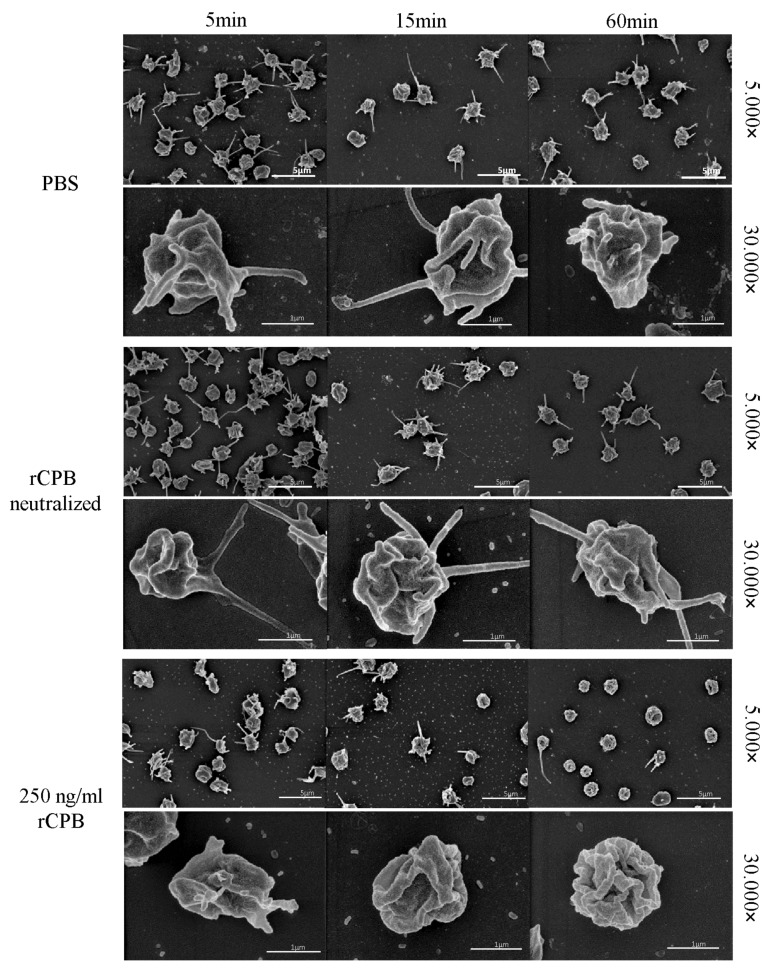
Analysis of the morphological effect of rCPB on porcine platelets using scanning electron microscopy. Platelets exposed to rCPB (250 ng/mL) showed rapid morphological changes compared to PBS-incubated platelets. Morphological effects were inhibited by antibody-mediated neutralization of rCPB. Scale bars: 5 μm (5.000×) or 1 μm (30.000×).

**Figure 4:**
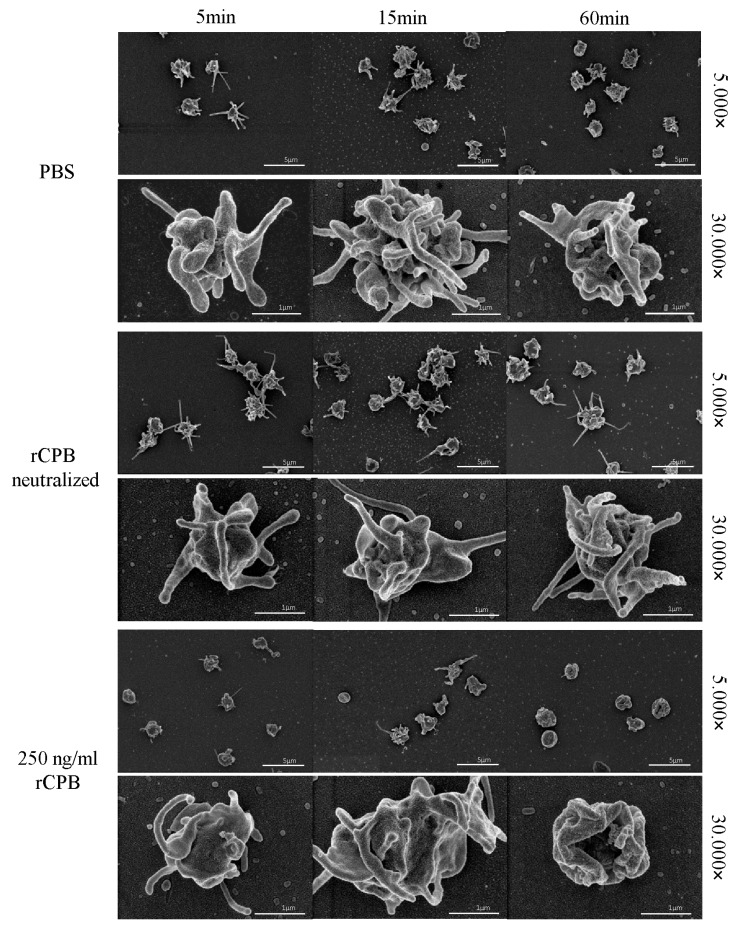
Morphological effect of rCPB on human platelets. Platelets exposed to rCPB (250 ng/mL) showed rapid morphological changes compared to PBS-incubated platelets. The morphological effect was inhibited by antibody-mediated neutralization of rCPB. Scale bars: 5 μm (5.000×) or 1 μm (30.000×).

In addition, porcine and human platelets showed early morphological changes when incubated with rCPB (Figure 3 and Figure 4). After 5 min of incubation with 250 ng/mL rCPB, a loss of the discoid platelet shape and formation of small pseudopodia was visible. After 15 min, porcine platelets showed rounding, while human platelets appeared swollen with pseudopodia entwining the platelet body. After 60 min of incubation, porcine and human platelets appeared shrunken and rounded with loss of pseudopodia compared to controls. 

Platelets incubated with neutralized rCPB or PBS (Figure 3 and Figure 4) showed formation of small pseudopodia but no signs of modification of the pseudopodia or the platelet shape over time. 

### 2.2. Platelet Binding and Oligomerization of rCPB

**Figure 5:**
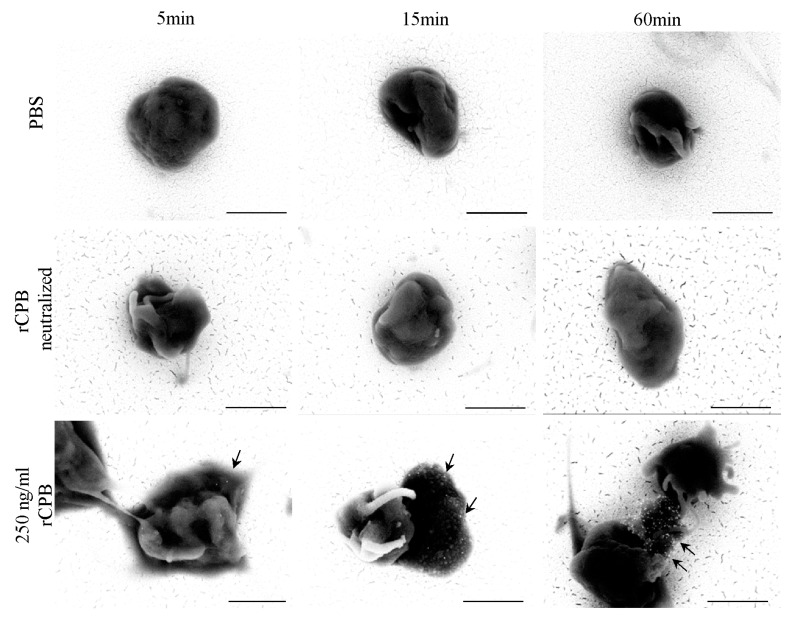
SEM backscattered electron image of rCPB bound on porcine platelets. Platelets were incubated with PBS, neutralized rCPB or rCPB (250 ng/mL) for indicated time points. Arrows indicate immuno-gold labeled rCPB. Scale bars: 1 μm, 30.000× magnification.

**Figure 6:**
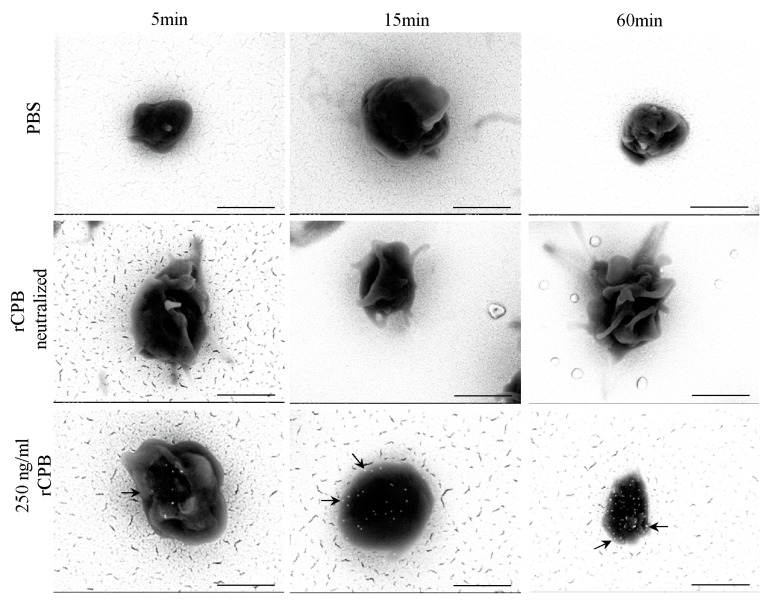
SEM backscattered electron image of rCPB bound on human platelets. Human platelets were incubated with PBS, neutralized rCPB or rCPB (250 ng/mL) for indicated time points. Arrows indicate immuno-gold labeled rCPB. Scale bars: 1 μm, 30.000× magnification.

To demonstrate the binding of rCPB on platelets, scanning electron microscopy (SEM) combined with immunogold labeling of rCPB was performed. Toxin (250 ng/mL)-treated platelets showed a signal of back-scattered electrons (BSE) of rCPB after 5 min of incubation and this signal increased at longer incubation times (Figure 5 and Figure 6). Controls, incubated with neutralized rCPB or PBS, remained unlabelled.

**Figure 7:**
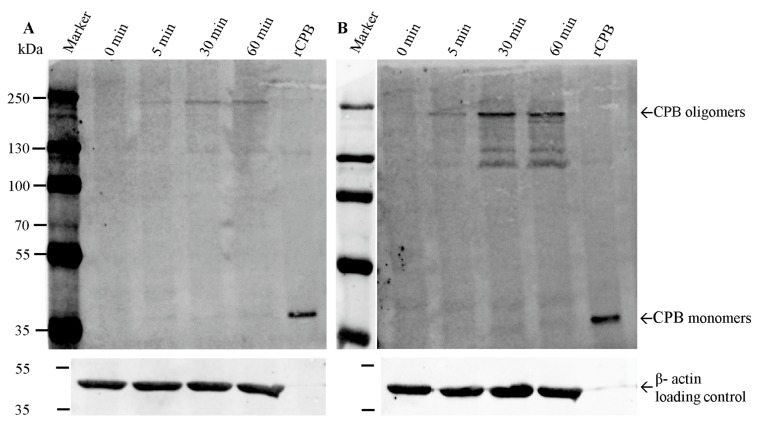
Binding and oligomerization of rCPB on porcine and human platelets. Porcine (**A**) and human (**B**) platelets were incubated with 1 μg/mL rCPB for indicated time periods, washed with PBS, lysed in RIPA buffer and boiled in SDS (Sodium Dodecyl Sulfate) loading buffer followed by SDS PAGE (Polyacrylamid Gel Electrophoresis) and Western blotting. A major band representing CPB oligomers located at a molecular mass of appr. 230 kDa. The monomeric form of CPB localizes at 35 kDa. Controls show purified monomeric rCPB and porcine and human platelet lysates without toxin incubation (0 min). Western blots were developed using monoclonal anti CPB and secondary antibody IRDye 680 CW.

Western blotting of RIPA(Radioimmunoprecipitation assay buffer) lysates of porcine and human platelets incubated with rCPB depicted, in addition to the 35 kDa monomeric form, that a prominent Western blot signal appeared at appr. 230 kDa (Figure 7), which corresponds to previously published CPB oligomers and an expected size of heptameric toxin complexes at the plasma membrane [4]. In addition, several high molecular weight bands between 220 kDa and appr. 130 kDa were present. The high molecular weight forms were demonstrated after 5 min of incubation of platelets with rCPB and the intensity of the band increased over time. 

### 2.3. Lack of Marked Platelet Activation by rCPB

**Figure 8:**
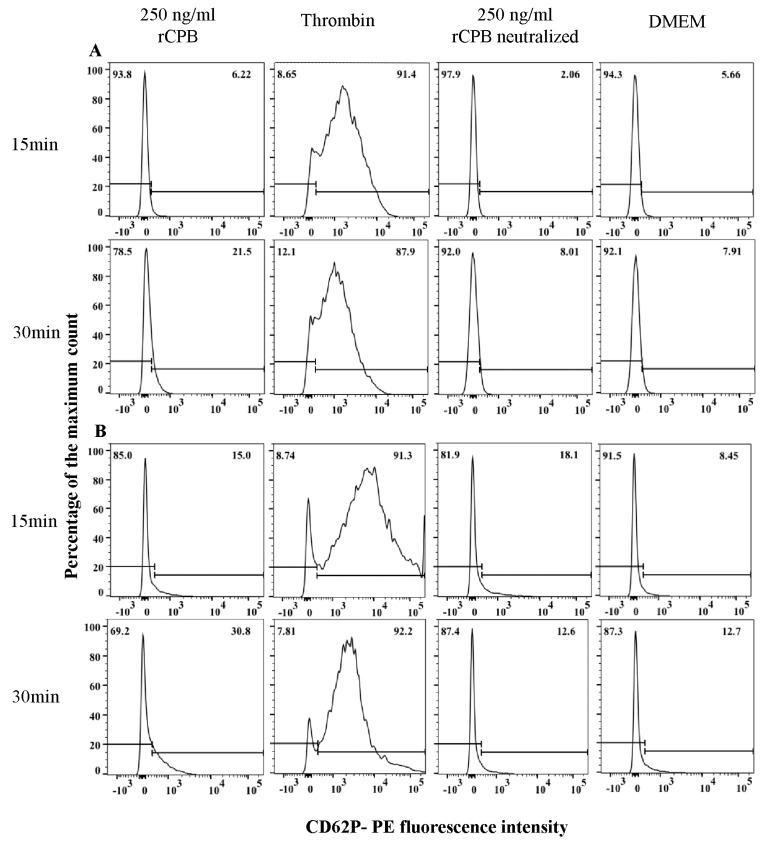
rCPB does not induce marked activation of porcine (**A**) and human (**B**) platelets. Platelets (33.3 × 10^6^/mL) were incubated with rCBP (250 ng/mL), thrombin (2.5 U), neutralized rCPB or DMEM for indicated time points. CD62P-PE labeled antibody was used as activation marker. The histograms show the Phycoerythrin (PE)-fluorescence intensity of porcine and human platelets, in which the PE positive fraction represents activated platelets.

As a marker for platelet activation, we investigated translocation of the platelet activation-dependent granule-external membrane protein CD62P to the plasma membrane by flow cytometry (Figure 8). Porcine and human platelets were incubated with rCBP (250 ng/mL), neutralized rCPB or DMEM for indicated time points. Incubation of porcine and human platelets with thrombin (2.5 U) served as a positive control and resulted in a >90% positive signal after 5 min and 15 min of stimulation. Control incubations with neutralized rCPB or DMEM showed weak positive signals with a maximum of appr. 12% positive platelets, whereas incubation with neutralized rCPB resulted in slightly higher percentages of positive platelets after 5 and 15 min of incubation. The signal of porcine and human platelets exposed to rCPB was slightly higher compared to negative controls but considerably weaker compared to thrombin activation.

### 2.4. Effect of rCPB on Platelet Function

**Figure 9:**
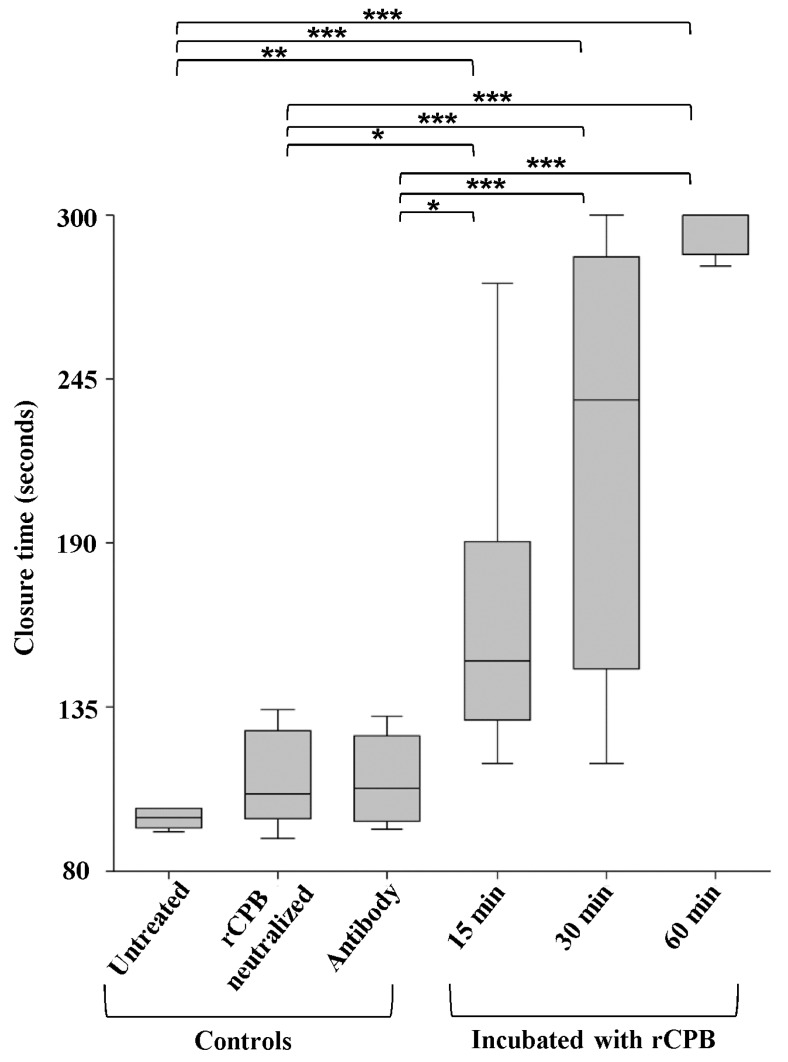
Exposure of whole human blood to rCPB inhibits platelet aggregation. Whole blood was incubated with rCBP (1 μg/mL) for indicated time points. Untreated blood or incubation with neutralized rCPB or monoclonal antibody 10A2 served as controls. ANOVA, Tukey–Kramer Multiple-Comparison Test. Asterisks describe the level of significance: * *p* < 0.05, ** *p* < 0.01, *** *p* < 0.001.

To assess whether CPB toxicity on platelets results in a loss of function, we performed a functional platelet aggregation assay established for human blood samples. This assay is performed with a platelet function analyser which aspirates whole blood into a test cartride through a small aperture of a membrane. The membrane is coated with collagen and adenosin diphosphat (ADP) and thus activates platelets to form a plug which closes the aperture. Addition of rCPB (1 μg/mL) to human blood samples followed by different incubation periods (15 min, 30 min or 60 min) led to a markedly prolonged aperture closure time of >140 s at 15 min and 30 min (Figure 9). At 60 min, no closure was achieved within the measurement interval of the test system, indicating failure of platelets to aggregate. Control samples were left untreated or incubated either with neutralized rCPB or the 10A2 antibody only. Closure times of all controls remained within the physiological range of >65 s to <130 s. The differences between the controls and rCPB-incubated groups were highly significant (ANOVA *p*-value < 0.05). The multiple comparisons Bonferronni-corrected tests showed that groups of controls (untreated blood, incubation with neutralized rCPB or antibody only) differed from the groups which were incubated with rCPB (15 min, 30 min and 60 min (Figure 9)). 

The box plot shows the closure time of the aperture by platelet plug formation. Asterisks indicate significant differences between groups. Whole blood incubated with rCPB for 15 min showed a significant prolonged closure time (*p* < 0.05) compared to untreated whole blood. The closure time was highly extended at 60 min (*p* < 0.001). 

**Figure 10:**
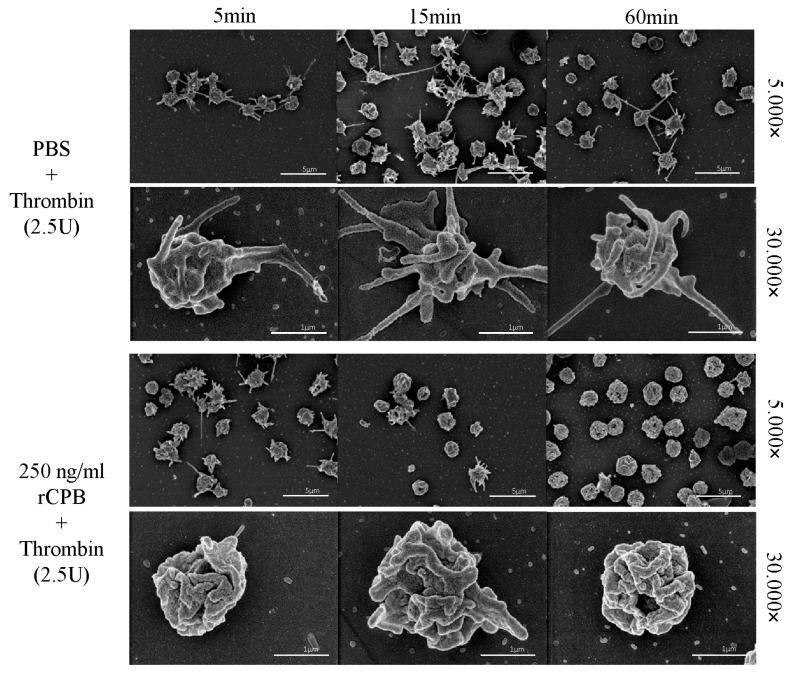
Porcine platelets exposed to rCPB are irresponsive to thrombin activation. Platelets were incubated with PBS (control) or rCPB for the indicated times followed by addition of thrombin. While control platelets reacted to thrombin with typical shape change and formation of pseudopodia at all time points, platelets incubated with rCPB (250 ng/mL) did not display such thrombin-induced changes.

**Figure 11:**
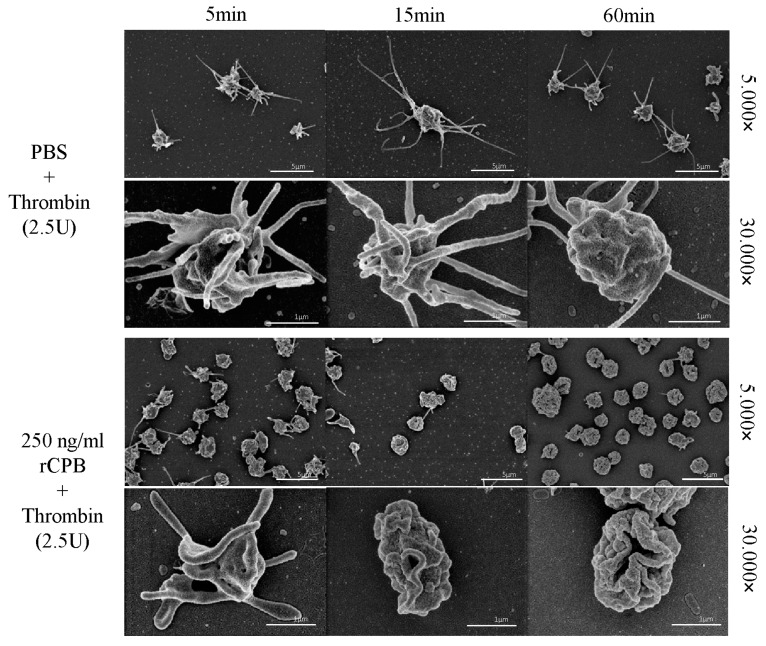
Human platelets exposed to rCPB are irresponsive to thrombin activation. Platelets were incubated with PBS or rCPB for the indicated times followed by addition of thrombin. While control platelets reacted to thrombin with typical shape change and formation of pseudopodia at all time points, platelets incubated with rCPB (250 ng/mL) showed only slight signs of activation at 5 min and did not display thrombin-induced changes after 15 or 60 min of toxin exposure.

In addition to this functional test, we evaluated whether platelets could still be activated after exposure to rCPB using electron microscopy. Addition of the platelet activator thrombin (2.5 U/mL) to PBS-treated platelets resulted in platelet activation with typical shape changes and formation of multiple pronounced pseudopodia. In contrast, toxin-treated platelets showed no morphological signs of activation by thrombin, even after 5 min of exposure to 250 ng/mL rCPB (Figure 10 and Figure 11).

## 3. Discussion

Our results demonstrate that CPB is highly toxic to porcine and human platelets. The cytopathic effect induced by CPB occurs very rapidly leading to morphological changes of platelets within a few minutes of exposure to the toxin. Furthermore, we demonstrated that CPB binds to porcine and human platelets and forms oligomers. Using scanning immune-electron microscopy, we confirmed the binding of CPB at the plasma membrane of porcine and human platelets. Affected platelets did not exhibit marked signs of primary activation, showed markedly reduced ability to aggregate and could not be activated by thrombin, indicating that CPB counteracts the physiological function of porcine and human platelets by inducing rapid cell death. We therefore propose that, in addition to endothelial cells, CPB targets platelets in vivo and has an inhibitory effect on primary hemostasis. This may explain the discrepancy between our previous observations in naturally infected piglets [15], a human case of Enteritis Necroticans [17] and an experimental infection model in newborn piglets [16]. Whereas thrombosis of mucosal vessels is a typical feature of end-stage lesions in naturally infected piglets, early histological lesions in an ileal loop model in newborn piglets were characterized by vascular leakage and hemorrhage without visible signs of early hemostasis by platelet aggregation and thrombus formation. Endothelial cell damage, as shown to be induced by CPB [9,10], would expose the subendothelial extracellular matrix during natural disease. This, in turn, would activate the hemostatic process [12,18,19] with adherence of platelets to vWF and collagen leading to platelet activation. Once activated, platelets change shape and release platelet agonists from their pre-formed granules (alpha and delta granules). These pro-thrombotic and pro-inflammatory mediators recruit and activate further platelets, leading to clot stabilization, retraction and thrombus formation. The targeting of platelets by CPB, as shown by our in vitro results, would lead to an impairment of primary hemostasis in vivo and could in part explain the massive and protracted intestinal hemorrhage observed in *C. perfringens* type C-induced enteritis [16]. Therefore, CPB may not only be responsible for early endothelial damage but also contribute to prolonged bleeding from damaged vessels. With regard to host–pathogen interaction, this would make sense, as it provides the bacterium with optimal growth conditions enabling *C. perfringens* to proliferate rapidly and even upregulate secretion of CPB and other toxins [20,21]. Once initiated, this would lead to a vicious cycle culminating in the segmental hemorrhagic necrosis of the small intestinal wall. A major role of CPB in the pathogenesis of *C. perfringens* type C enteritis therefore is the induction of hemorrhage during the early phases of the disease process. 

Our results indicate that porcine platelets were slightly more susceptible to rCPB than human platelets, despite the fact that oligomers formed in both platelet types. This difference has not been detected so far in human and porcine endothelial cells [9,10]. Possible reasons are species-specific characteristics of platelets. It is well known that differences in receptor expression exist together with differences in granule content [22,23,24]. Composition and quantity of coagulation factors are also influenced by the species, which plays an important role in the hemostatic process [22,25]. In-depth studies of the membrane interactions of CPB with porcine and human target cells would be required to investigate this difference. Nevertheless, human platelets were still highly susceptible to CPB and showed very similar reaction patterns as porcine platelets. In conclusion, our results add platelets to the list of target cells for CPB and extend the current hypothesis of the role of this toxin in the pathogenesis of *C. perfringens* type C enteritis.

## 4. Materials and Methods

### 4.1. Isolation of Platelets

Pig blood was collected at the Institute of Virology and Immunology, Mittelhäusern, Switzerland, and was approved by the cantonal veterinary office. Porcine platelets were isolated from citrated blood of specific pathogen-free pigs using different centrifugation steps (1st: 1.000 *g*, 20 min, 4 °C; 2nd: 2.500 *g*, 10 min, 4 °C). Human platelets were obtained from apheresis filters from the Blood Transfusion Center, Bern (project nr. 129). Donor blood was tested according to the Swiss regulations for the preparation of blood and blood components (Heilmittelgesetz, HMG). The study was conducted in accordance with the Declaration of Helsinki. All subjects gave their informed consent for inclusion of anonymized biological material derived from their blood samples in research studies. Due to anonymization of all blood samples, our study was not regulated by the Swiss Human Research Law (HFG). Human platelets were isolated by centrifugation (1st: 600 *g*, 20 min, 4 °C; 2nd: 2.500 *g*, 10 min, 4 °C). Platelets were counted using the MUSE Cell Analyzer (Merck Millipore, Merck KGaA, Darmstadt, Germany), diluted as required for the subsequent experiments and stored at 4 °C in Dulbecco’s Modified Eagle Medium (DMEM, Gibco, Paisley, UK) containing 10% fetal calf serum (FCS) or in Dulbecco’s Phosphate Buffered Saline (PBS, Gibco, Paisley, UK) until further processing.

### 4.2. Incubation of Platelets with Recombinant Clostridium Perfringens β-Toxin

Recombinant CPB (rCPB) was produced as previously described [10]. Thrombin, which was used for rCPB production, was removed using streptavidin-coated beads. Platelets were incubated with rCPB at 37 °C. As negative controls, platelets were incubated in DMEM or PBS without addition of toxin or rCPB was neutralized with anti CPB monoclonal antibody 10A2 (100 μL/mL, Center for Veterinary Biologics, Ames, IA, USA) for 30 min at room temperature (RT). Incubation of platelets with 2.5 U thrombin (Sigma-Aldrich, Buchs, Switzerland) was used as a control for activated platelets. 

### 4.3. Flow Cytometry

Isolated porcine and human platelets were transferred to 5 mL FACS (fluorescence-activated cell sorting) tubes at 5 × 10^6^ platelets per tube and incubated with 10 and 250 ng/mL (porcine) or 50 and 250 ng/mL (human) rCPB, neutralized rCPB or DMEM (15, 30, 60 and 120 min). After incubation, platelets were washed with PBS and centrifuged at 700× *g* for 5 min at 4 °C. For viability staining platelets were resuspended in PBS, stained with Calcein-AM (1 μM; Sigma-Aldrich Chemie GmbH, Buchs, Switzerland) for 30 min at 37 °C and analyzed using a BD FACS Canto II flow cytometer (BD Biosciences, Allschwil, Switzerland). Data were analyzed using FlowJo software 8 (Tree Star Inc., Ashland, OR, USA). For the determination of platelet activation, porcine and human platelets were incubated with rCPB (250 ng/mL) or thrombin (2.5 U, Sigma-Aldrich, Buchs, Switzerland). After incubation (15 and 30 min) platelets were washed with PBS and centrifuged at 700× *g* for 5 min at 4 °C to remove unbound rCPB. Labelling was performed with anti-CD62 antibodies (clone Psel.KO.2.5, Bio-Rad, USA or clone AK4, Lubio Science GmbH, Zurich, Switzerland) for 30 min on ice followed by washing with PBS, centrifugation and analyzation with BD FACS as described above.

### 4.4. Scanning Electron Microscopy 

Porcine and human platelets (1 × 10^9^/mL) were incubated with rCPB (250 ng/mL), neutralized rCPB or PBS for 5, 15 and 60 min. Thereafter, 2.5U of thrombin were added to samples immediately after incubation for 1 min. After washing with PBS and centrifugation at 700× *g* for 5 min, samples were fixed with 2.5% glutaraldehyde (Merck, Darmstadt, Germany) in 0.1 M cacodylate buffer, pH 7.2, for 45 min at RT. Fixed samples were centrifuged at 125× *g* for 5 min in a cytospin centrifuge (Hettich Universal 320, Tuttlingen, Germany) onto gold-sputtered and Poly-l-Lysin-coated (Sigma P8920, 0.1%) coverslips. Postfixation was done with 2% OsO_4_ (Polysciences, Warrington, PA, USA) in 0.1 M cacodylate buffer for 30 min. After two washes in 0.1 M cacodylate buffer, the samples were dehydrated through an ascending ethanol series and dried by evaporation of Hexamethyldisilazane (Merck, Schaffhausen, Switzerland) as described previously [26,27]. Afterwards, the coverslips were mounted on metal stubs with conductive adhesive tabs (Ted Pella, Redding, CA, USA) and sputter-coated with approximately 20 nm of gold with an SCD004 (BalTec, Balzers, Liechtenstein). Secondary electron micrographs were obtained with a field emission scanning electron microscope DSM 982 Gemini (Zeiss, Oberkochen, Germany) at an accelerating voltage of 5 kV at a working distance of 5–8 mm and a magnification of 30.000×. Binding of rCPB to platelets was investigated by immuno-scanning electron microscopy. Porcine and human platelets (1 × 10^9^/mL) were incubated with rCPB (250 ng/mL), neutralized rCPB or PBS for 5, 15 and 60 min at RT. After washing with PBS and centrifugation at 700× *g* for 5 min, samples were fixed with 4% buffered formaldehyde (Methanol-free, Thermo Scientific, Waltham, MA, USA) for 30 min at RT. Fixed samples were centrifuged at 125× *g* for 5 min in a cytospin centrifuge onto gold-sputtered and Poly-l-Lysin-coated coverslips as described above. After incubation with glycine (0.05 M) combined with 0.1% bovine serum albumin (BSA, blocking agent, Merck, Darmstadt, Germany) in PBS for 15 min at RT, platelets were washed with PBS/BSA. Afterwards platelets were incubated with a 1st anti-*β*-toxin antibody (10A2, 1:10) over night at 4 °C and a 2nd colloidal gold-labeled antibody (18 nm, goat anti mouse igG antibody, Jackson ImmunoResearch, West Grove, PA, USA) for 90 min at RT. Postfixation was done with 2% OsO_4_ (Polysciences, Warrington, PA, USA) in 0.1 M cacodylate buffer for 30 min. After two washes in 0.1 M cacodylate buffer, the samples were dehydrated through an ascending ethanol series and dried by evaporation of hexamethyldisilazane as described above.

### 4.5. Detection of Toxin Oligomerization by Western Blot Analyses 

Porcine and human platelets (3.2 × 10^9^/mL) were incubated with rCPB (1 μg/mL) for 0, 5, 30 and 60 min, followed by washing with PBS (centrifugation at 700× *g* for 10 min at 4 °C), to remove unbound rCPB. After lysis in 100 μL RIPA-buffer (50 mM Tris-HCL, pH 7.4, 150 mM NaCl, 1% NP-40, 0.25% Na-deoxycholate, 1 mM EDTA and 0.1% SDS), lysates were centrifuged (15,294× *g*, 10 min, 4 °C). After boiling in SDS sample buffer (Laemmli buffer), equal amounts of proteins were separated by a 7% SDS gel and blotted on a nitrocellulose membrane (Trans-Blot Turbo Transfer Pack, Bio-Rad, Hercules, CA, USA). Western blots were developed using the mouse anti-CPB monoclonal antibody 10A2 (1:1.000) and a donkey anti-mouse secondary antibody IRDye 680 CW (1:6.000 dilution, Li-Cor Biosciences). As a loading control, identical volumes of samples were separated by 7% SDS-PAGE, blotted on nitrocellulose membranes and developed using anti-actin antibodies (1:5.000 dilution, anti-*β*-actin antibody, Sigma) followed by a donkey anti-mouse secondary antibody IRDye 680 CW (1:6.000 dilution). Signals were detected and visualized using the Azure c600 imaging system (Azure Biosystems, Inc., Dublin, CA, USA).

### 4.6. Functional Assay

Platelet function was evaluated with the platelet function assay (Siemens Healthcare Diagnostics Products GmbH, Marburg, Germany), based on previous publications [28,29]. Shortly, this tests the process of platelet adhesion and aggregation following a simulated vascular injury in vitro. Citrated whole blood is aspirated from the sample reservoir through a collagen- and ADP-coated capillary with a small aperture, which exposes platelets to shear stress and two activating factors. During the test, functional platelets adhere to the membrane, become activated form aggregates to build a thrombus at the aperture, thereby gradually reducing the blood flow. The platelet function analyzer system determines the time from the start of the test until the platelet plug occludes the aperture, and reports this time interval as the closure time [30]. The physiological values for samples from healthy human blood donors ranges between 65 and 130 s. Citrated whole blood (3.8 mL) was provided by volunteer employees of the authors’ institute. All subjects gave their informed consent for inclusion of anonymized biological material derived from their blood samples in research studies and the study was conducted in accordance with the Declaration of Helsinki. Blood samples were incubated with rCPB (1 µg/mL) at RT for 15, 30 and 60 min. Control samples were left untreated or incubated either with neutralized rCPB or the 10A2 antibody only. For each condition, 10 samples were measured. Statistics were done using the NCSS software. At 60 min, often no closure was achieved within the measurement interval of the test system, but for statistic calculations, the value was set at 300 s, the maximum value of the test system. Differences between the treated groups were tested by means of analysis of variance (ANOVA) with Bonferroni (All-Pairwise) multiple-comparison test (the level of significance, alpha, was set to alpha 0.05).

## References

[1] Peraro M.D., van der Goot F.G. (2016). Pore-forming toxins: Ancient, but never really out of fashion. Nat. Rev. Microbiol..

[2] Popoff M.R., Bouvet P. (2009). Clostridial toxins. Future Microbiol..

[3] Sayeed S., Uzal F.A., Fisher D.J., Saputo J., Vidal J.E., Chen Y., Gupta P., Rood J.I., McClane B.A. (2008). Beta toxin is essential for the intestinal virulence of *Clostridium perfringens* type C disease isolate cn3685 in a rabbit ileal loop model. Mol. Microbiol..

[4] Nagahama M., Hayashi S., Morimitsu S., Sakurai J. (2003). Biological activities and pore formation of clostridium perfringens beta toxin in HL 60 cells. Biol. Chem..

[5] Nagahama M., Shibutani M., Seike S., Yonezaki M., Takagishi T., Oda M., Kobayashi K., Sakurai J. (2013). The p38 mapk and jnk pathways protect host cells against *Clostridium perfringens* beta-toxin. Infect. Immun..

[6] Seike S., Takehara M., Kobayashi K., Nagahama M. (2016). Role of pannexin 1 in *Clostridium perfringens* beta-toxin-caused cell death. Biochim. Biophys. Acta (BBA) Biomembr..

[7] Steinthorsdottir V., Halldorsson H., Andresson O.S. (2000). *Clostridium perfringens* beta-toxin forms multimeric transmembrane pores in human endothelial cells. Microb. Pathog..

[8] Autheman D., Wyder M., Popoff M., D’Herde K., Christen S., Posthaus H. (2013). *Clostridium perfringens* beta-toxin induces necrostatin-inhibitable, calpain-dependent necrosis in primary porcine endothelial cells. PLoS ONE.

[9] Popescu F., Wyder M., Gurtner C., Frey J., Cooke R.A., Greenhill A.R., Posthaus H. (2011). Susceptibility of primary human endothelial cells to *C. perfringens* beta-toxin suggesting similar pathogenesis in human and porcine necrotizing enteritis. Vet. Microbiol..

[10] Gurtner C., Popescu F., Wyder M., Sutter E., Zeeh F., Frey J., von Schubert C., Posthaus H. (2010). Rapid cytopathic effects of *Clostridium perfringens* beta-toxin on porcine endothelial cells. Infect. Immun..

[11] Roos S., Wyder M., Candi A., Regenscheit N., Nathues C., van Immerseel F., Posthaus H. (2015). Binding studies on isolated porcine small intestinal mucosa and in vitro toxicity studies reveal lack of effect of *C. perfringens* beta-toxin on the porcine intestinal epithelium. Toxins.

[12] Li Z., Delaney M.K., O’Brien K.A., Du X. (2010). Signaling during platelet adhesion and activation. Arterioscler. Thromb. Vasc. Biol..

[13] Davie E.W., Fujikawa K., Kisiel W. (1991). The coagulation cascade: Initiation, maintenance, and regulation. Biochemistry.

[14] Zhao Q., Li H., Li B. (2011). Nanoencapsulating living biological cells using electrostatic layer-by-layer self-assembly: Platelets as a model. J. Mater. Res..

[15] Miclard J., Jaggi M., Sutter E., Wyder M., Grabscheid B., Posthaus H. (2009). *Clostridium perfringens* beta-toxin targets endothelial cells in necrotizing enteritis in piglets. Vet. Microbiol..

[16] Schumacher V.L., Martel A., Pasmans F., Van Immerseel F., Posthaus H. (2013). Endothelial binding of beta toxin to small intestinal mucosal endothelial cells in early stages of experimentally induced *Clostridium perfringens* type C enteritis in pigs. Vet. Pathol..

[17] Miclard J., van Baarlen J., Wyder M., Grabscheid B., Posthaus H. (2009). *Clostridium perfringens* beta-toxin binding to vascular endothelial cells in a human case of enteritis necroticans. Med. Microbiol..

[18] Versteeg H.H., Heemskerk J.W.M., Levi M., Reitsma P.H. (2013). New fundamentals in hemostasis. Phys. Rev..

[19] Watson S.P. (2009). Platelet activation by extracellular matrix proteins in haemostasis and thrombosis. Curr. Pharm. Des..

[20] Chen J., Ma M., Uzal F.A., McClane B.A. (2014). Host cell-induced signaling causes *Clostridium perfringens* to upregulate production of toxins important for intestinal infections. Gut Microbes.

[21] Vidal J.E., Ohtani K., Shimizu T., McClane B.A. (2009). Contact with enterocyte-like Caco-2 cells induces rapid upregulation of toxin production by *Clostridium perfringens* type C isolates. Cell. Microbiol..

[22] Lutze G., Lutze G., Kutschmann K., Wiens L. (2007). Plasma blood coagulation in mammals (domestic and zoo animals). Experience with screening tests and determinations of individual factor activities. Hämostaseologie.

[23] Gruba S.M., Koseoglu S., Meyer A.F., Meyer B.M., Maurer-Jones M.A., Haynes C.L. (2015). Platelet membrane variations and their effects on δ-granule secretion kinetics and aggregation spreading among different species. Biochim. Biophys. Acta (BBA) Biomembr..

[24] Pelagalli A., Belisario M.A., Tafuri S., Lombardi P., d’Angelo D., Avallone L., Staiano N. (2003). Adhesive properties of platelets from different animal species. J. Comparat. Pathol..

[25] Softeland E., Framstad T., Thorsen T., Holmsen H. (1992). Porcine platelets in vitro and in vivo studies: Relevance to human thrombosis research. Eur. J. Haematol..

[26] Stoffel M.H., Friess A.E. (2002). Demonstration and cytochemical analysis of anionic sites on ejaculated boar spermatozoa: A scanning electron microscopy study using cationised colloidal gold. Histochem. Cell Biol..

[27] Stoffel M.H., Busato A., Friess A.E. (2002). Density and distribution of anionic sites on boar ejaculated and epididymal spermatozoa. Histochem. Cell Biol..

[28] Kratzer M.A.A., Born G.V.R. (1985). Simulation of primary haemostasis in vitro. Pathophysiol. Haemost. Thromb..

[29] Kratzer M.A.A., Bellucci S., Caen J.P. (1985). Detection of abnormal platelet functions with an in vitro model of primary haemostasis. Pathophysiol. Haemost. Thromb..

[30] Kundu S.K., Heilmann E.J., Sio R., Garcia C., Ostgaard R.A. (1996). Characterization of an in vitro platelet function analyzer, pfa-100™. Clin. Appl. Thromb./Hemost..

